# Preexisting compensatory amino acids compromise fitness costs of a HIV-1 T cell escape mutation

**DOI:** 10.1186/s12977-014-0101-0

**Published:** 2014-11-19

**Authors:** Donglai Liu, Tao Zuo, Bhavna Hora, Hongshuo Song, Wei Kong, Xianghui Yu, Nilu Goonetilleke, Tanmoy Bhattacharya, Alan S Perelson, Barton F Haynes, Andrew J McMichael, Feng Gao

**Affiliations:** Duke Human Vaccine Institute, Duke University Medical Center, Durham, NC 27710 USA; National Engineering Laboratory For AIDS Vaccine, College of Life Science, Jilin University, Changchun, 130012 Jilin China; Department of Microbiology, Immunology and Medicine, University of North Carolina at Chapel Hill, Chapel Hill, NC 27599 USA; Theoretical Division, Los Alamos National laboratory, Los Alamos, NM 87545 USA; Weatherall Institute of molecular Medicine, University of Oxford, Oxford, OX3 9DS England UK

**Keywords:** HIV-1, Fitness, Immune escape mutation, Compensatory mutation, Cytotoxic T lymphocytes, Transmitted/founder virus, Reversion

## Abstract

**Background:**

Fitness costs and slower disease progression are associated with a cytolytic T lymphocyte (CTL) escape mutation T242N in Gag in HIV-1-infected individuals carrying HLA-B*57/5801 alleles. However, the impact of different context in diverse HIV-1 strains on the fitness costs due to the T242N mutation has not been well characterized. To better understand the extent of fitness costs of the T242N mutation and the repair of fitness loss through compensatory amino acids, we investigated its fitness impact in different transmitted/founder (T/F) viruses.

**Results:**

The T242N mutation resulted in various levels of fitness loss in four different T/F viruses. However, the fitness costs were significantly compromised by preexisting compensatory amino acids in (Isoleucine at position 247) or outside (glutamine at position 219) the CTL epitope. Moreover, the transmitted T242N escape mutant in subject CH131 was as fit as the revertant N242T mutant and the elimination of the compensatory amino acid I247 in the T/F viral genome resulted in significant fitness cost, suggesting the fitness loss caused by the T242N mutation had been fully repaired in the donor at transmission. Analysis of the global circulating HIV-1 sequences in the Los Alamos HIV Sequence Database showed a high prevalence of compensatory amino acids for the T242N mutation and other T cell escape mutations.

**Conclusions:**

Our results show that the preexisting compensatory amino acids in the majority of circulating HIV-1 strains could significantly compromise the fitness loss due to CTL escape mutations and thus increase challenges for T cell based vaccines.

## Background

Soon after HIV-1 infection, hosts mount potent CTL responses [1-3]. However, HIV-1 can quickly develop resistance by generating escape mutations to mitigate the potency of the CTL responses [2-4]. These CTL escape mutants may have reduced viral fitness and lowered viral loads which can lead to slower disease progression or reduced probabilities of transmission to new hosts [5-10]. A recent study showed dominant viral quasispecies with higher *in vivo* fitness levels had an overall transmission advantage and even marginally reduced viral fitness might lower the overall transmission rates and offer long-term benefits upon successful transmission [11]. However, the better clinical outcome may not be sustained into chronic infection [10], possibly due to subsequent compensatory mutations that occur both within and outside the targeted epitopes and repair the fitness loss [10,12-16].

The T242N mutation in the HLA-B*57/5801-restricted TW10 epitope in Gag p24^240–249^ (TSTLQEQIGW) has been widely studied for its impact on viral fitness, pathogenesis, and disease progress [8,10,14,15] as well as the repair of its fitness costs by compensatory mutations in heterologous viral backbones [7,14,15,17]. However, the fitness costs of the T242N and other T cell escape mutations have been exclusively studied in unrelated heterologous viral backbones, in which the presence of unknown compensatory mutations can significantly affect the interpretation of results. Moreover, since fitness costs of the T242N mutation have not been studied in transmitted/founder (T/F) viral genomes, it remains unknown how the context of different T/F genomes affect its fitness impact, especially in the cognate T/F viruses from which the T242N mutation was selected or in the transmitted T242N mutants. Studies have shown that T cell escape mutations with little or no fitness costs can be prevalent in the HLA matched and mismatched populations [18] or persist for a long time without reverting back to the wild type [19,20]. Thus, the increased prevalence of the CTL escape mutations consequent on preexisting compensatory amino acids at the population level may further reduce benefits of the protective HLA alleles to slow HIV disease progression, posing challenges for T cell based vaccines.

Using an unmodified infectious T/F molecular clone (CH77) from which the T242N was selected *in vivo*, we previously reported that the T242N mutation had a significant fitness cost in its cognate T/F virus but the fitness loss could be fully repaired by the compensatory mutations in the same TW10 epitope [21,22]. To fully understand the impact of the T242N mutation on viral fitness, we now investigated its fitness costs and the compensation in four additional T/F viruses.

## Results and discussion

### The CTL escape mutation T242N caused various levels of fitness loss in different virus backbones

**Figure 1:**
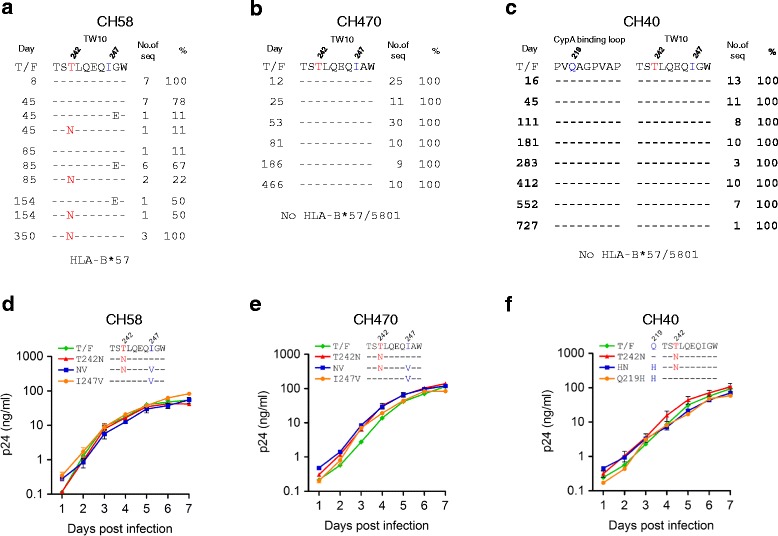
**Replication kinetics of T cell escape mutants.** Frequencies of mutations in the TW10 epitope or CypA binding loop were determined by comparing the longitudinal sequences to the T/F sequences for CH58 **(a)**, CH470 **(b)**, and CH40 **(c)**. The days post screening are indicated. The T242N escape mutation and compensatory amino acids were introduced into the CH58 **(d)**, CH470 **(e)**, and CH40 **(f)** T/F viral genomes. The replication kinetics of the mutants and their corresponding T/F viruses were determined by measuring p24 concentrations in the cell culture supernatants. The amino acids associated with T cell escape at the position 242 are indicated in red while the amino acids associated with compensation of the fitness loss at the positions 219 and 247 are indicated in blue. Each virus was cultured in triplicate. Mean values ± standard deviations are shown.

The T242N mutation was first detected 45 days post screening and was fixed in the viral population at day 350 in patient CH58 who was positive for HLA-B*57, which presents TW10 (Figure 1a), while it was not selected in CH470 and CH40 (Figure 1b and c), neither of which carry HLA-B*57/5801 alleles [3,4]. A G248E mutation was also detected at the same time when the T242N mutation occurred in CH58. However, it became undetectable while the T242N mutation was fixed in the viral population at day 350. Our previous study showed that the T242N mutation resulted in a significantly reduced T cell response while the G248E mutation only modestly affected T cell responses. The G248E mutation, however, abrogated binding of KIR3DL1 to HLA-B*5703, suggesting the emergence of this mutation was more likely associated with NK cell recognition [23]. Since the G248E was not clearly associated with T cell escape, it existed only transiently in the viral population, and the focus of the currently study was to determine fitness costs of the T242N mutation in different genetic backgrounds, the G248E mutation was not further studied.

**Figure 2:**
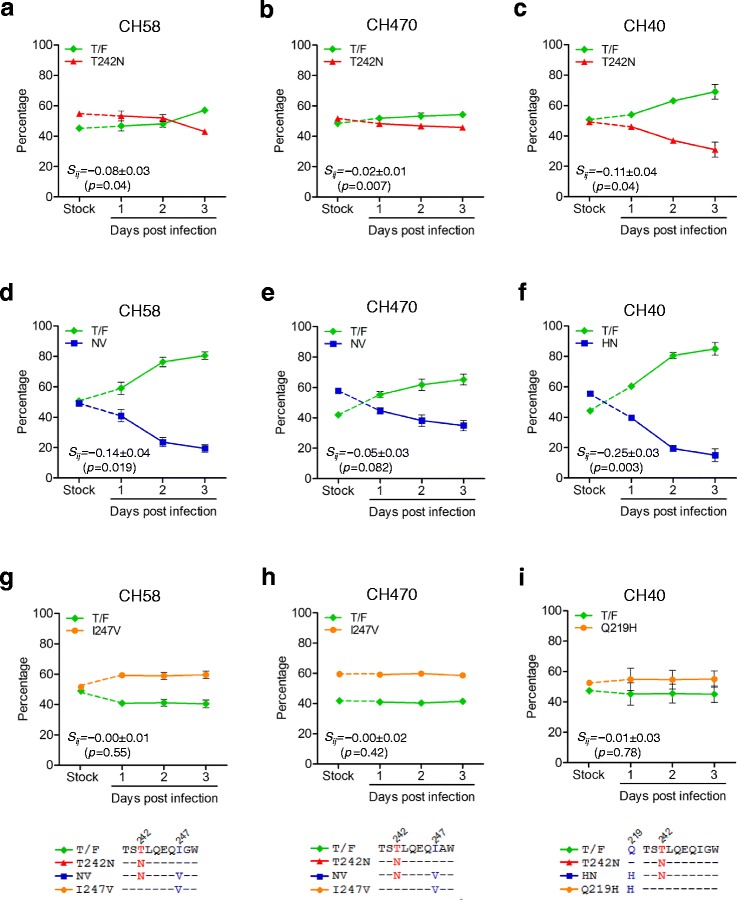
**Fitness loss caused by the T242N mutation was partially restored by the preexisting compensatory amino acids.** The same amount (2.5 ng p24) of the T/F and its mutant was mixed to infect freshly purified CD4^+^ T cells, and the proportion of each virus in the culture was determined by the PASS fitness assay. The relative fitness of the T242N mutants was determined for CH58 **(a)**, CH470 **(b)**, and CH40 **(c)** by comparing each T242N mutant to its corresponding T/F virus. The increased fitness loss of the T242N mutants after removing the compensatory amino acids was determined for CH58 **(d)**, CH470 **(e)**, and CH40 **(f)** by comparing each double-mutation virus to its corresponding T/F virus. The impact on fitness in the T/F viruses by the compensatory amino acids alone was determined by introducing those amino acids in the corresponding T/F viral genomes for all three viruses: CH58_I247V_ **(g)**, CH470_I247V_ **(h)** and CH40_Q219H_ **(i)**.

To determine whether the T242N mutation could cause similar fitness loss in different viral backbones, we introduced it into these three T/F genomes. All three T242N mutants replicated as well as their corresponding T/F viruses when the T/F and T242N mutant viruses were cultured independently (Figure 1d-f). We then determined their relative fitness by growing both the T242N mutant and its corresponding T/F virus in the same culture and determining the proportion of each virus over time using the parallel allele-specific sequencing (PASS) method, in which two compared viruses (wild type and mutant) were grown in the same cell culture, and proportions of the viruses at different time points were determined by directly detecting the wild type and mutant bases in the viral genomes through analysis of a large number of viruses in the culture supernatant [21,22]. In all three comparisons, the T242N mutants were outgrown by the corresponding T/F viruses over time in the culture (Figure 2a-c). Using the mathematical model that we developed earlier [21,22], we found that all three T242N mutants were significantly less fit than their corresponding T/F viruses (p = 0.04 for CH58_T242N_ and CH40_T242N_; p = 0.007 for CH470_T242N_). However, the fitness losses caused by the T242N mutation were different among these three T242N mutants. It was 11 ± 4% less fit for CH40_T242N_ and 8 ± 3% less fit for CH58_T242N_, but only 2 ± 1% less fit for CH470_T242N_ (Figure 2a-c). These results demonstrated that the T cell escape mutation T242N could decrease the viral fitness in different T/F viruses but the levels of fitness loss varied in the different virus backbones.

The difference of the Malthusian growth parameters characterizing two viral strains is not the only factor that controls competition. For example, a virus that can infect a cell fast and prevent superinfection will show an advantage in a competitive assay, even if its overall growth rate is not high. In addition, the estimates of Malthusian parameters often have rather large errors, of order 5% or more, due to variations in the growth rate seen among replicate experiments. Since this level of variation is similar to the competitive advantage that has been observed in many cases, to reveal a fitness advantage of this magnitude, we would need to grow the virus in replicate experiments under almost identical conditions. In practice, since one cannot control the conditions that accurately between replicates, one needs to simultaneously grow the viruses one wishes to compare.

### The fitness loss due to the T242N mutation could be compromised by the preexisting compensatory amino acids

Examination of the TW10 epitope sequences in these three viruses showed that they all had Isoleucine (Ile) at position 247 (Figure 1a-c), which could compensate the fitness loss caused by the T242N mutation in CH77 but neither I247 nor V247 alone had detectable fitness impact on the cognate T/F virus [22]. The V247I mutation was selected by T cell responses before the T242N T cell escape mutation in CH77 which had Val at position 247. It subsequently served as a compensatory mutation to significantly restore the fitness loss caused by the T242N mutation that was selected by later T cell responses [22]. To determine if the preexisting I247 also mitigated the T242N mutation’s impact in these T/F viruses, we removed this potential compensatory amino acid by introducing the I247V mutation into each T242N mutant to generate double mutant viruses (NV). Both CH58_NV_ and CH470_NV_ replicated similarly to their T/F viruses and T242N mutants (Figure 1d and e). However, CH40_NV_ completely lost its replicative capacity as reported for some other lethal mutations in the replication competent HIV-1 genome [24,25]. Examination of the cyclophilin A (CypA) binding loop sequences showed that the glutamine at position 219, which can compensate fitness costs due to the T242N mutation [7,14,26,27], was present only in the CH40 T/F virus. Therefore, we introduced the Q219H mutation into CH40_T242N_ to generate double mutant CH40_HN_, which also replicated as well as its T/F virus and T242N mutant (Figure 1f). When compared to the corresponding T/F viruses, more fitness loss were observed for all three double mutant viruses; CH58_NV_, CH470_NV_ and CH40_HN_ were 14 ± 4%, 5 ± 3% and 25 ± 3% less fit than the corresponding T/F viruses, respectively (Figure 2d-f). These results showed that the T242N mutants without the compensatory amino acids were significantly less fit than the corresponding T/F viruses and the T242N mutants with the compensatory amino acids.

To determine if the compensatory amino acids alone could cause fitness loss and contributed to the increased fitness loss in the NV or NH double mutants, we generated T/F mutants that contained the compensatory amino acids but not the T242N mutation (the I247V mutation in CH58 and CH470 as well as the Q219H in CH40; Figure 1d-f). All three mutants (CH58_I247V_, CH470_I247V_ and CH40_Q219H_) replicated similarly as their corresponding T/F viruses (Figure 1d-f). When they were cultured together with their corresponding T/F viruses, CH58_I247V_, CH470_I247V_ and CH40_Q219H_ did not change their proportions with the T/F viruses throughout the culture (Figure 2g-i). These results indicated that the compensatory amino acids alone had no impact on the viral fitness and that the increased fitness loss in double mutants (CH58_NV_, CH470_NV_ and CH40_HN_) was indeed due to the removal of compensatory amino acids. Taken together, our data demonstrated that the fitness costs caused by the T242N mutation could be significantly mitigated by preexisting compensatory amino acids.

### The transmitted CTL escape T242N mutant had no significant fitness costs

**Figure 3:**
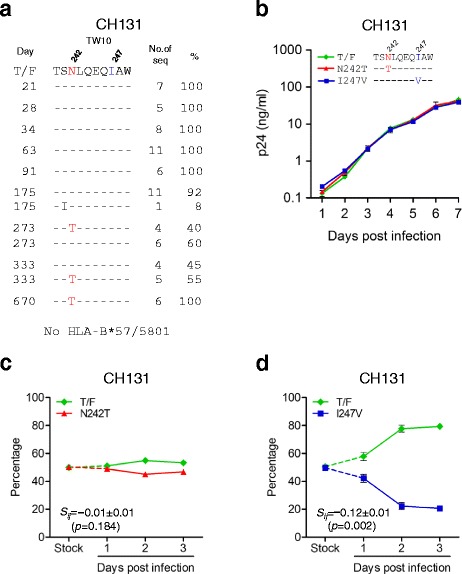
**Fitness impact of the T242N mutation and its compensatory amino acid in the transmitted T242N escape mutant.** **(a)** The frequency of the T242N escape mutation in the TW10 epitope was determined by comparing the longitudinal sequences to the CH131 T/F sequence. The days post screening are indicated. **(b)** The N242T reversion mutation and I247V mutation were introduced into the CH131 T/F viral genome and the replication kinetics of the mutants and their corresponding T/F virus were determined by measuring p24 concentrations in the cell culture supernatants. **(c)** The relative fitness of the T242N reversion mutant was determined by comparing to the corresponding T/F virus by the PASS fitness assay. **(d)** The fitness cost of the T242N mutant was determined by comparing the I247V mutant that contained the T242N mutation but not the compensatory isoleucine at position 247 to the T/F virus.

The transmitted CTL escape mutants carrying the T242N mutation were thought to be less fit than the wild type viruses [7,8,10]. However, the T242N escape mutant viruses were frequently transmitted into new hosts and its reversion back to the wild type generally took months or years [7,10,27,28]. These observations suggested that the T242N mutants might not be less fit than the wild type viruses. However, the fitness impact of the T242N mutation has not been determined in the cognate transmitted T242N escape mutant. CH131 was infected with a T242N escape mutant and did not have the HLA-B*57/5801 alleles [4]. The reversion of the T242N mutation was first detected at day 273 and was fixed at day 670 (Figure 3a). To determine whether the T242N reversion mutation (N242T) could render the transmitted T242N escape mutant more fit, we generated the N242T reversion mutant by substituting N242 with T242 in the CH131 T/F viral genome and compared its fitness with the CH131 T/F virus. The reversion mutant CH131_N242T_ replicated as well as the T/F virus when cultured independently (Figure 3b). When co-cultured with the CH131 T/F virus, no significant fitness difference was observed between CH131 T/F and CH131_N242T_ viruses (Figure 3c). To test whether the absence of detectable fitness costs of the T242N mutation in the T/F virus was due to the presence of compensatory I247, we generated the CH131_I247V_ mutant by introducing the I247V mutation in the T/F viral genome (Figure 3b). Indeed, CH131 without the compensatory amino acid I247 (CH131_I247V_) was 12 ± 1% less fit than the T/F virus (p = 0.002) (Figure 3d).

These results demonstrated that the impaired fitness of the transmitted T242N escape mutant was fully repaired by the compensatory amino acid I247. Thus, the T/F virus, although carrying a fitness-impairing T cell escape mutation, could be as infectious and transmissible as the wild type virus. Since the reversion of the T242N to the wild type did not have any impact on the fitness of the escape mutant, the reversion of the T242N escape mutation would take a long time to occur in CH131 and as reported in other studies [7,10,27,28]. These results also suggested that the reversion of preexisting T cell escape mutations whose fitness loss was well compensated by the compensatory amino acids might not affect viral fitness.

### The fixation of fitness-impairing T242N mutation did not lead to the viral load decrease

**Figure 4:**
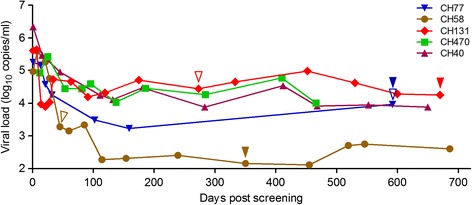
**Association of the T242N mutation with the viral load.** The viral load set points were stabilized from ~100 days after screening in five HIV-1 infected subjects. The T cell escape mutation T242N was selected *in vivo* in subjects CH77 and CH58. CH131 was infected with the T242N escape mutant. The time for the first detection of the T242N escape mutation (blue and brown) or the N242T reversion mutation (red) is indicated by open triangle and the time for the fixation of the T242N or N242T mutation in the viral population is indicated by solid triangle. No T242N mutation was detected in CH40 and CH470.

A small fitness difference may cause significant change in viral populations [29]. Thus, the significant fitness cost of the T242N mutation could have caused a significant viral load (VL) decrease if it was fixed in the viral population. However, no VL decrease was observed in CH77 and CH58 after the T242N escape mutation was fixed in the viral population (Figure 4). Such an absence of VL decrease in CH77 might be due to the complete repair of the fitness loss by the compensatory mutations [21,22]. However, VL did not decreased in CH58 either, although the T242N escape mutant was significantly less fit than the T/F virus (Figure 4). Similar lack of VL decrease was also observed in other studies [4,30]. This pervasive lack of association between the fixation of the T242N mutation and the VL decrease suggested that the significant fitness costs were generally compensated during fixation. The slow fixation of the reversion mutation T242N in the viral population in CH131 (397 days between the first detection and the fixation) (Figure 4) and other viruses [7,10,27,28] lends further support to the view that the fitness cost of the T242N mutation was well compensated.

### The high prevalence of compensatory amino acids in the global circulating HIV-1 viruses could readily compromise the fitness loss caused by the T cell escape mutations

**Figure 5:**
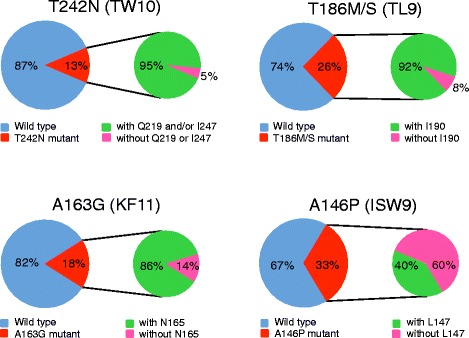
**The prevalence of the T cell escape mutations in T cell epitopes with or without compensatory amino acids.** One sequence from each HIV-1 infected individual in the Los Alamos HIV-1 Sequence Database was analyzed for four well-studied T cell epitopes (TW10, TL9, KR11 and ISW9). Over 4000 sequences were analyzed for each epitope. The frequency of the sequences with the CTL escape mutations and the frequency of the CTL mutant sequences with and without the compensatory amino acids were determined for each CTL epitope.

I247 is present in the vast majority (94%) of the circulating virus sequences in the Los Alamos HIV sequence database. Thus, the clinical benefits of the T242N escape mutation could be mitigated significantly since the predominance of the compensatory amino acid can readily counteract the fitness loss of the T242N escape mutants in those individuals, as shown in a recent study in which the high population-level frequency of compensatory mutations for T cell escape mutation in TW10 and KF9 epitopes were found associated with limited protective effect of the B*5801 allele [31]. The T242N mutation is present in 13% of viruses in the Los Alamos HIV sequence database (Figure 5), suggesting that the T242N CTL escape mutant is frequently transmitted and does not revert back quickly. Interestingly, the T242N mutation is almost exclusively (95%) found linked to Q219 and/or I247 in the database (Figure 5). Similar results were obtained for the escape mutations in three other CTL epitopes (TL9, KF11 and ISW9) for which the compensatory mutations were characterized [12,32-35] (Figure 5).

The presence of T cell escape mutations and their compensatory amino acids in a large proportion of HIV-1-infected population, especially in the individuals without restricting HLA alleles, suggested that viruses that escaped from the T cell responses but had its fitness loss restored by compensatory amino acids could successfully transmit to new hosts with or without restricting alleles. Since the transmitted viruses with both T cell escape mutations and their compensatory amino acids could be as fit as the wild type viruses, It will be unlikely for them to quickly revert back to the wild type sequences and result in the high prevalence of the CTL escape mutations and compensatory amino acids in large proportions of the general viral population. If escape mutations accumulate at a high frequency and become predominant (or consensus), it may not be possible to observe their effect [36]. Thus, this effect can be best seen when HLA alleles are examined with lower frequencies. However, the fact that we observed this in four alleles is consistent with this not being a rare phenomenon.

## Conclusions

**Figure 6:**
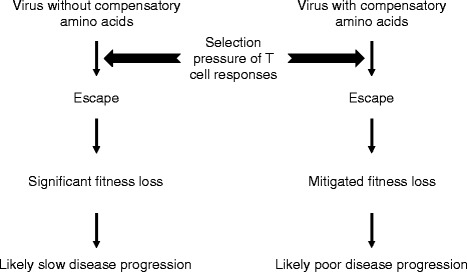
**Interaction among host immune selection, escape, viral fitness and potential disease progression.**

We demonstrated that the fitness loss caused by the T242N mutation could be significantly affected by the differing context of viral backbones because of preexisting compensatory amino acids that either were present in the general viral population (CH40, CH470 and CH58) or selected by immune responses (CH77) [21,22]. Thus, high prevalence of the compensatory amino acids could significantly mitigate the benefits of the fitness costs caused by the T242N escape mutation (Figure 6). These results indicate that true fitness costs of T cell escape mutations will be better revealed by analyzing those escape mutations in the context of their cognate T/F viral genomes or in multiple viral backbones when the infectious cognate T/F clones are not available. Our data also shows that the potential clinical benefits of T cell escape mutations should not be analyzed independent of compensatory mutations. More importantly, the fitness compensation effects on the T cell escape mutations by those preexisting compensatory amino acids should be considered for the design of T cell based vaccines. If the fitness loss caused by a T cell escape mutation can be readily restored by the preexisting compensatory amino acids or mutations, the vaccine-elicited T cell responses may not control such viruses as effectively as T cell escape mutant viruses that contain no compensatory amino acids or mutations to restore fitness loss.

Importantly, the transmitted T242N escape mutant could be as fit as the wild type virus, suggesting its fitness loss could have been fully repaired by the compensatory amino acids in the donor at the transmission as observed in CH77 [21,22]. With little or no fitness costs, some CTL escape mutants could be as transmissible and pathogenic as the wild type viruses. More importantly, since those T cell escape mutant viruses have little or no fitness costs, they will not revert back to wild type and can persist for a long time in the newly infected population [19,20]. As a result, the T cell escape mutations can be present at high percentages in the HLA matched and mismatched populations (Figure 5) [18]. The high level prevalence of the CTL escape mutations may reduce the benefits of the protective HLA alleles to slow HIV disease progression and pose challenges for T cell based vaccines. The implication for T cell vaccines is that T cell responses, including in those who share HLA alleles and induce T cell responses targeting the same epitopes, may not affect fitness costs uniformly among vaccinees. Thus, Immunogen designs that consider fitness costs of T cell escape in the context of the entire HIV-1 protein [37-39] may prove more efficacious.

## Methods

### Site-directed mutagenesis

Infectious molecular clones (IMCs) for CH40, CH58, CH470, and CH131 T/F viruses were generated in previous studies [40,41]. Mutations at positions 219, 242, 247 in Gag were introduced into the T/F IMCs by using the QuikChange II Site-Directed Mutagenesis Kits (Stratagene, Santa Clara, CA). All final mutant clones were sequence confirmed. The viral stocks were generated by transfecting the IMCs into 293 T cells as previous described [42].

### Viral replication kinetics

Peripheral blood mononuclear cells (PBMC) were obtained through leukophereses from healthy donors under clinical protocols approved by the Duke University Institutional Review Board. PBMCs were isolated using the Ficoll-Hypaque density gradients and lymphocytes were isolated by elutriation using standard techniques. CD4^+^ T cells were negatively selected from PBMCs or lymphocytes on an QuadroMACS Separator (Miltenyi Biotec, Auburn, CA) using the CD4^+^ T cell Isolation Kit II (Miltenyi Biotec, Auburn, CA). Fresh purified CD4^+^ T cells were stimulated for three days in RPMI1640 containing 10% fetal bovine serum (FBS), interleukin 2 (IL-2) (32 IU/ml; Advanced Biotechnologies, Columbia, MD), soluble anti-CD3 (0.2 μg/ml; eBioscience, San Diego, CA) and anti-CD28 (0.2 μg/ml; BD Bioscience, San Diego, CA). After stimulation, 50 μl of cell suspension (5 × 10^5^ cells) was seeded into each well of a 96-well plate, and infected with individual virus (500 TCID_50_) at 37°C for 4 hours. The cells were then washed three times with RPMI 1640 and cultured in a 48-well plate with 300 μl of RPMI1640 containing 10% FBS and IL-2 (32 IU/ml). The culture supernatant (50 μl) was harvested daily and the same volume fresh medium was replenished. The viral replication was monitored by measuring the p24 concentration in the culture supernatant using the Alliance HIV-1 P24 ANTIGEN ELISA Kit (PerkinElmer, Waltham, MA). All infections were performed in triplicate.

### PASS fitness assay

CD4^+^ T cells (5 × 10^5^) were infected with the mixture of two compared viruses (2.5 ng of p24 for each virus). The culture supernatant was harvested daily by completely replacing the culture medium. The viral replication kinetics was monitored by determining the p24 concentration in the supernatant. The culture supernatants were first treated with DNase (New England Biolabs Inc., MA, US) at 37°C for 20 minutes. Viral RNA (vRNA) was extracted from 50–100 μl of the treated culture supernatants and eluted into 20 μl of RNase free water using the PureLink Viral RNA/DNA Mini Kit (Invitrogen, Carlsbad, CA). vRNA (17 μl) was used for cDNA synthesis using SuperScript III reverse transcriptase (Invitrogen, Carlsbad, CA) with the primer lower3 (5’-TTTTTCCTAGGGGCCCTGCAATTT-3’; nt 1998–2021 in HXB2). The percentage of each virus in the culture supernatants was determined as previously described [21,43]. Briefly, 20 μl of 6% acrylamide gel mix, containing 1 μM acrydite-modified reverse primer M6F2 (5’-Acry-CTCGACGCAGGACTCGGCTTGCTG-3’; nt 685–708), viral cDNA, 0.3% diallyltartramide, 5% Rhinohide, 0.1% ammonium persulfate (APS), 0.1% N,N,N′,N′-tetramethylethylenediamine (TEMED) and 0.2% bovine serum albumin (BSA), was used to cast the gel on a bind-saline (Amersham Biosciences, Piscataway, NJ) treated glass slide. The in-gel PCR amplification was then performed in a PTC-200 Thermal Cycler with a mix of 1 μM forward primer M6R2 (5’-TCCTCCCACTCCCTGACATGCTGTCATCATTTC-3’; nt 1822–1854), 0.1% Tween-20, 0.2% BSA, 1xPCR buffer, 100 μM dNTP mix, 3.3 units of Jumpstart Taq DNA polymerase (Sigma, St. Louis, MO), and H_2_O (up to 300 μl) under a sealed SecurSeal chamber (Grace Bio-Labs, Inc., Bend, OR). The partial *gag* gene (1164 bp-1183 bp) was amplified. The PCR conditions were 94°C for 3 min; 65 cycles of 94°C for 30 sec, 56°C for 45 sec, and 72°C for 3 min; and 72°C for 6 min. After in-gel PCR, the gels were treated with denaturation solution to remove the free DNA strands. Single base extension (SBE) was then performed to distinguish the compared viruses using two different labeled bases with sequencing primers that annealed just upstream of the target sites. To detect the bases at position 242, sequencing primer CH40/58_TW10 (5’-CATCCATCCTATTTGTTCCTGAAGG-3’; nt 1515–1539) was used for CH40 and CH58, and sequencing primer CH470_TW10: (5’-CATCCATGCTATTTGTTCCTGAAGG-3’; nt 1515–1539) for CH470 and CH131. To detect the bases at the compensatory amino acid sites, sequencing primer CH58_247 (5’-ATAGGTGGATTACTTGTCATCCATCCTA-3’; nt 1529–1556) and CH470_247 (5’-ATAGGTGGATTATTTGTCATCCATGCTA-3’; nt 1529–1556) were used for determining position 247 in CH58 and CH470, respectively; CH40_219 (5’-TCATCTGGCCTGGTGCAACAGGCCCTGC-3’; nt 1447–1474) for determining position 219 in CH40; CH131_247 (5’-GGTGGGTTACTTGTCATCCATGCTA-3’; nt 1529–1553) for CH131. After SBE, the gel was scanned to acquire images with the GenePix 4000B Microarray Scanner (Molecular Devices, Sunnyvale, CA).

The two channel images (Cy5 and Cy3 for the bases from the two compared virus templates, respectively) were first cropped with Picture Window Pro3.5 (Digital Light & Color, Belmont, MA) to remove the edge area containing no specific signals. The cropped images were then analyzed with the Progenesis PG200 software (Nonlinear Dynamics, Durham, NC). After background subtraction, normalization, and spot filter setting, only unambiguous spots at both channels were included for further analysis. The normalized pixel count data at two mutation sites at each spot were exported into an Excel file with a unique identifier. By comparing each spot’s normalized values at both channels, the different viruses were identified based on the base identity, and the percentage of each compared virus in the viral population was then determined as previous described [21,22,43].

### Model for relative fitness

The relative fitness (*Sij*) is determined in competitive assays by measuring the relative replication slope of the viruses in culture over time using the mathematical model described previously [21,22]. There are two sources of statistical uncertainty in the measurement of slope: one arising from the binomial fluctuations expected of the counts in the PASS assay, and the other due to the differences between the three replicates of each competition assay. In general, the inter-experiment differences were much larger than the binomial estimation uncertainty. We, therefore, quote this error, and use Student’s t-test to assess the significance of the measured fitness difference. All the statistical analyses were done using the statistical package R [44] with the subplex [45] and ucminf [46] libraries to do the parameter estimations and using the gplots [47] library for visualization.

## Abbreviations

CTL: Cytolytic T lymphocyte
T/F: Transmitted/founder
PASS: Parallel allele-specific sequencing
CypA: Cyclophilin A
VL: Viral load
IMC: Infectious molecular clone
